# Phantom for standardization in functional near-infrared spectroscopy, part 2: optical properties and Monte Carlo simulations

**DOI:** 10.1117/1.NPh.13.1.015015

**Published:** 2026-03-11

**Authors:** Caterina Amendola, Antonio Pifferi, Alessandro Torricelli, Dirk Grosenick, Heidrun Wabnitz, David R. Busch, Michele Lacerenza, Marianne Floor-Westerdijk, Sanathana Konugolu Venkata Sekar, Claudia Nunzia Guadagno, Yukari Tanikawa, Hiroshi Kawaguchi

**Affiliations:** a Politecnico di Milano, Department of Physics, Milan, Italy; b Istituto di Fotonica e Nanotecnologie, Consiglio Nazionale delle Ricerche, Milan, Italy; c Physikalisch-Technische Bundesanstalt (PTB), Berlin, Germany; d University of Texas Southwestern Medical Center, O’Donnell Brain Institute, Department of Anesthesiology, Neurology, and Biomedical Engineering, Dallas, Texas, United States; e PIONIRS s.r.l., Milan, Italy; f Artinis Medical Systems, Elst, The Netherlands; g BioPixS Limited, Cork, Ireland; h Tyndall National Institute, Cork, Ireland; i University College Cork, School of Physics, Cork, Ireland; j National Institute of Advanced Industrial Science and Technology (AIST), Tsukuba, Japan

**Keywords:** functional near-infrared spectroscopy, phantom, international standard, absorption, scattering

## Abstract

**Significance:**

The international standard IEC 80601-2-71 defines requirements for the basic safety and essential performance of medical continuous wave functional near-infrared spectroscopy (fNIRS) equipment, including a set of tests based on a dedicated tissue-equivalent phantom consisting of switchable apertures sandwiched between two diffusing media.

**Aim:**

In a companion paper, we described a first implementation of this phantom developed by AIST, its basic characterization of attenuation properties, and results of its application in the testing of commercial fNIRS devices. In the present paper, we provide the absorption and reduced scattering spectra of the AIST phantom and of alternative silicone phantom matrices, complemented with extensive simulations on the effect of the phantom optical properties and geometry on the features of the phantom relevant for the fNIRS standard test.

**Approach:**

All spectral measurements were performed with time-domain diffuse optical spectrometers for accurate quantification of the absorption and reduced scattering coefficient, whereas simulations were obtained using a Monte Carlo code.

**Results:**

The POM-C matrix of the AIST phantom exhibits negligible absorption ($<0.01\;{\mathrm{cm}}^{-1}$) and a monotonically decreasing reduced scattering coefficient (from 10.5 down to $8.5\;{\mathrm{cm}}^{-1}$) in the 650 to 850 nm range. Monte Carlo simulations explain the independence from wavelength, geometry, and partially also optical properties in the change in attenuation when switching between two apertures of 6 and 4 mm diameter within the phantom, with <0.1  dB spectral variation. Extended spectral characterization of POM-C and silicone matrices in the 600 to 1100 nm range showed additional marked absorption peaks around 900 and 1020 nm.

**Conclusions:**

We provide valid support for researchers and manufacturers to understand the operation of the phantom described in the fNIRS standard and to replicate it in their labs. In addition, we provide a basis for further evolution of the standard itself to encompass alternative probe geometries or functional tests.

## 1 Introduction

Functional near-infrared spectroscopy (fNIRS) is an optical method to noninvasively assess changes in oxy- and deoxyhemoglobin in the brain cortex associated with cerebral activation (due to, e.g., cognitive, visual, or motor stimuli).^1^^,^^2^ Alteration in the absorption spectrum of hemoglobin when it binds oxygen and relatively low absorption of biological tissues in the 650 to 900 nm range allows one to track cerebral activation by measuring attenuation of light between injection and collection points on the scalp at a few wavelengths. Several different approaches have been realized that use continuous wave (CW), time-domain (TD), or frequency domain (FD) light illumination and detection.^3^ The applications of fNIRS encompass basic neuroscience research, psychopedagogical studies, neuroeconomics, brain–computer interface, sport, and leisure.^4^ Clinically approved commercial devices have been deployed, initially in Japan, and are used in neurosurgical preoperative testing and to assist in the differential diagnosis of depressive symptoms.^5^^,^^6^

The international standard IEC 80601-2-71 defines requirements for the basic safety and essential performance of medical CW fNIRS equipment. It was developed by the IEC/ISO Joint Working Group (JWG) “Oximeters” (IEC/TC 62/SC 62D/JWG 5 and ISO/TC 121/SC 3/JWG 10). In 2025, following a systematic review, the second edition of this standard was published.^7^ Several performance tests for fNIRS equipment in this standard are based on a dedicated phantom that is equipped with a mechanism to change the light intensity detected by the fNIRS device under test. Although the major specifications of the phantom are defined in the standard, its specific technical implementation is not detailed there. Therefore, it was advisable to provide fNIRS manufacturers with information about a valid example of the practical implementation of this phantom. In 2022/2023, during the revision of this standard, an international measurement campaign was organized among members of the JWG “Oximeters.” It was led by the National Institute of Advanced Industrial Science and Technology (AIST) and was carried out as a round-robin campaign, circulating one of the original phantom prototypes developed at AIST. The participants included various European fNIRS manufacturers and research institutions that were interested in gaining experience with the phantom prototype and its application in fNIRS device testing. The development of the fNIRS standard has previously been reported.^8^

In a companion paper,^9^ the fNIRS standard phantom developed by AIST (hereafter briefly called AIST phantom) was presented together with the basic characterization of its attenuation properties and results of its application in the testing of commercial fNIRS devices. The goal of the present paper is to help researchers and manufacturers understand the requirements of the fNIRS standard and replicate the tests in their labs. More specifically, we provide: (i) the absorption ($\mu_{a}$) and reduced scattering ($\mu_{s}^{\prime }$) spectra of the AIST phantom in the range 600 to 850 nm; (ii) a Monte Carlo (MC) study of the effect of the optical properties and geometrical parameters of the phantom on its total attenuation and on the magnitude of the attenuation change, which are key requirements of the fNIRS standard; (iii) the extended spectral characterization in the 600 to 1100 nm range of suitable materials to construct a fNIRS standard phantom.

Implications of the findings for the implementation of the fNIRS tests will be presented. Furthermore, the paper will address some subtleties in the accurate characterization of phantom materials, particularly when dealing with low absorption and finite dimensions.

## 2 fNIRS Phantom

The international standard for fNIRS equipment, IEC 80601-2-71, describes tests to evaluate the performance of CW fNIRS devices. Measuring changes in detected light intensities is a key capability of CW fNIRS devices; therefore, the fNIRS standard prescribes a phantom test to assess this capability. The standard defines the basic specifications of the phantom, i.e., ranges for the absorption coefficient ($\mu_{a}$), reduced scattering coefficient ($\mu_{s}^{\prime }$), and size, for example. White polyoxymethylene copolymer (POM-C) is recommended as the main turbid material for the phantom due to the similarity of its $\mu_{s}^{\prime }$ to biological tissues. The $\mu_{a}$ of white POM-C is much lower than that of biological tissues and is almost constant in the wavelength range of the light sources used by fNIRS devices. In addition, POM-C has excellent mechanical and processing properties. The fNIRS standard does not prevent the use of epoxy resin or silicone resin, which are often applied as the base material of traditional fNIRS phantoms. Further characterizations of the various materials will be presented in Sec. 3.

The AIST phantom meets the specifications of the standard and consists of three main parts: an emission side attenuator, a detection side attenuator, and a circular aperture of changeable size sandwiched between the two attenuators. The two attenuators are cylindrical pieces of POM-C with a diameter of 60 mm and thicknesses of 20 and 10 mm on the injection and detection sides, respectively. The light is injected and detected at the centers of the circular surfaces. The light travels between the two attenuators through the aperture only. The active aperture is 4 or 6 mm in diameter, small compared with the diameter of the attenuators, and can be easily mechanically exchanged. The center of the active aperture is designed to coincide with the central axis of the cylindrical POM-C attenuators. By switching one aperture to the other, the detected light intensity is expected to change approximately proportionally to the areas of the apertures, i.e., by a factor of 2.25. See the part 1 paper for more details.^9^ Replicas of the prototype became commercially available from a phantom manufacturer (BioPixS Ltd., Cork, Ireland), which is one of the major outcomes of this work.

## 3 Materials and Methods

The AIST phantom was characterized at AIST using a CW spectrometer to provide high spectral resolution and a TD diffuse spectrometer at Physikalisch-Technische Bundesanstalt (PTB, Germany) to separate absorption from scattering contribution. An extended spectral characterization of POM-C and silicone for phantom construction was performed with TD diffuse spectrometers at Politecnico di Milano (POLIMI, Italy) and Tyndall National Institute (TYNDALL, Ireland), respectively. POLIMI conducted MC simulations on different phantom implementations. Each institution adopted the best practices for measurement procedures and data analysis established in their lab, as detailed below.

### 3.1 CW Spectroscopy

A POM-C slice from the same bulk as the fNIRS standard phantom (Duracon NE25X, Polyplastics, Tokyo, Japan) was cut into a rectangular shape of 40×40×5  mm. The surface roughness (Ra) of the sample was 0.8. The sample was measured with a spectrophotometer equipped with an integrating sphere (UV-2600i and ISR-2600Plus for UV-2600, Shimadzu, Kyoto, Japan). The widest surface of the sample was held in close contact with the aperture of the integrating sphere to measure diffuse transmittance and reflectance. The measurement wavelength range was 650 to 900 nm, and automatic sweeps were repeated seven times at 1 nm intervals to calculate the mean and standard deviation for each wavelength. To check the effect of bias due to different sample attachment positions, the sample was attached and removed five times and measured in the same manner, and the mean was taken.

### 3.2 TD Diffuse Spectroscopy

The PTB time-domain system utilizes a SuperK Fianium FIU-15 PP supercontinuum laser combined with a SuperK Varia tunable filter (NKT Photonics, Germany) for wavelength selection. The pulse repetition rate was set to 39 MHz. To estimate the optical properties of the turbid blocks of the fNIRS standard phantom, the 2 cm thick POM-C cylinder (diameter 60 mm) was taken out of the housing of the phantom and placed between two plates. These plates were covered with black Neoprene and contained holes to hold the ferrules of optical fibers. The measurements were performed in transmission geometry. A graded index fiber of 62.5  μm core diameter was used to guide the laser pulses to the center of the front face of the cylinder. On the rear face, the transmitted light was collected on axis by a graded-index fiber with a core diameter of 600  μm. The distributions of times of flight of photons (DTOF) were recorded by time-correlated single photon counting (TCSPC) with a cooled fast hybrid detector (HPM-100-07C, Becker & Hickl, Germany) and an SPC-150NX board (Becker & Hickl, Germany). A variable neutral density filter in front of the detector was employed to adjust the photon count rate.

The measurements on the cylinder were done at wavelengths from 600 to 845 nm in steps of 10 nm, with a spectral bandwidth of ∼10  nm. The average light power on the surface of the phantom was a few milliwatt at each wavelength. Using an automatic wavelength sweep with 25 s steps, a collection time interval of 20 s was available for analysis at each wavelength. The maximum photon count rate over all wavelengths was adjusted not to exceed 500 kHz. The full width at half maximum (FWHM) of the instrument response function (IRF) was ∼50  ps at 600 nm and decreased with increasing wavelength to ∼35  ps in the 800 to 845 nm range. For each wavelength, the measured DTOF was analyzed by a white MC model (e.g., simulations were performed with $\mu_{a}$ set to zero, and absorption effects were added a posteriori according to Beer’s law) of light transport in a homogeneous cylinder. For this purpose, a database of DTOFs with a step size of $\Delta \mu_{s}^{\prime }=0.1\;{\mathrm{cm}}^{-1}$ was created, with $\mu_{a}$ set to zero. DTOFs for intermediate values of $\mu_{s}^{\prime }$ were determined by linear interpolation. When fitting the optical properties, absorption was taken into account by multiplying the DTOFs of the database by the Lambert Beer factor $\exp(-\mu_{a}c_{M}t)$ where $c_{M}$ and t denote the speed of light in the phantom and the time of flight, respectively. In addition to $\mu_{a}$ and $\mu_{s}^{\prime }$, a small temporal offset between the theoretical DTOF (convolved with the IRF) and the measured DTOF was used as a third fitting parameter. Including the offset in the fitting improves the matching of the DTOF’s leading edge by the model and also corrects for a possible temporal drift between the phantom and IRF measurements. After analyzing the DTOFs at each wavelength independently, the $\mu_{s}^{\prime }$ were smoothed by applying the scattering power law

$$\mu_{s}^{\prime }(\lambda )=\mu_{s}^{\prime }(\lambda_{0})(\lambda /\lambda_{0}{)}^{-b},$$ (1)

with $\lambda_{0}$ set to 800 nm. Then, $\mu_{a}$ and the temporal offsets were fitted a second time to obtain the final result for absorption.

The POLIMI system^10^ operates a supercontinuum white light laser source (SuperK Extreme, NKT Photonics, Denmark) emitting pulses with a width on the order of picoseconds at 40 MHz repetition rate as an illuminating source. Spectral scanning of the source wavelength was achieved by first dispersing the white laser light using a Pellin Broca prism (Bernhard Halle Nachfl, Germany) placed on a rotating stage for wavelength tuning, and then selecting a narrow band by a small core (50  μm) graded index fiber, resulting in a spectral bandwidth of 3 to 9 nm over the 600 to 1100 nm wavelength range. After attenuation by a variable neutral density filter (Edmund Optics, Barrington, New Jersey, United States), light was injected into and collected from the phantom by a pair of 1 mm core, 1.5 m long glass step-index fibers in reflection geometry. Light gathered from the phantom at a source-detector distance of 2 cm was delivered to a home-made detection module based on a silicon photomultiplier (SiPM) (S10362-11-050C, Hamamatsu, Japan) with custom driving electronics.^11^ The DTOF of re-emitted photons was recorded using a TCSPC board (SPC-130, Becker & Hickl, Berlin, Germany). All processes involved in the spectral measurement (wavelength scanning, laser attenuation, data visualization, and saving) were automated by a custom ANSI C software implemented in the LabWindows CVI framework. A full spectrum, from 600 to 1100 nm at steps of 5 nm, required around 5 min. The dark background of the instrument was $2\cdot 10^{4}$ counts per second, and the FWHM of the IRF was around 90 ps. The maximum power impinging onto the sample was 4 mW, whereas the count rate was kept $<10^{6}$ counts per second. The absorption and reduced scattering spectra were derived by fitting the DTOFs to the convolution of the IRF with a solution of the photon diffusion equation for a cylindrical geometry. Only $\mu_{a}$ and $\mu_{s}^{\prime }$ were used as free-fitting parameters, whereas the time origin was defined from the IRF.

The TYNDALL system is based on a supercontinuum broadband laser source (Fianium SC450, Southampton, United Kingdom) with a 20 MHz repetition rate and pulse width in the tens of picoseconds range. White light was dispersed using a rotatable Pellin Broca prism (UV Fused Silica, Thorlabs, Newton, New Jersey, United States, ADBU-20). The wavelength selection was achieved by coupling light into a graded index fiber (0.1 NA, 25  μm, M67L02, Thorlabs) using a convex lens. The fiber tip, which was in contact with the sample, acted as a source. Due to the nonlinear dispersion of the prism, the FWHM of the selected light wavelength band varied with wavelength, ranging from 5 nm at 650 nm to 12 nm at 1100 nm. A multimode fiber (0.39 NA, 300  μm, M67L02, Thorlabs) was employed for light collection in transmission geometry. The detection system included a single-photon avalanche diode (SPAD, Micro Photon Devices, Bolzano, Italy, PDM freespace) and custom-made front-end optics to focus the light onto the 100  μm active area. The detector’s signal was processed by a laser-synchronized TCSPC board (Picoharp 300, PicoQuant GmbH, Berlin-Adlershof, Germany), which acquired and stored the DTOF data for further analysis. To control irradiance at the sample and prevent detector saturation, a pair of continuously variable neutral density filters (NDC-100C-4, Ø100 mm, OD: 0.04 to 4.0, Thorlabs) was used, ensuring photon count rates remained within acceptable limits to avoid pulse pile-up. The aim was to maintain a count rate of $∼3\cdot 10^{5}$ counts per second, which is <2% of the laser repetition rate.

### 3.3 Monte Carlo Simulations of Phantom Properties

**Fig. 1:**
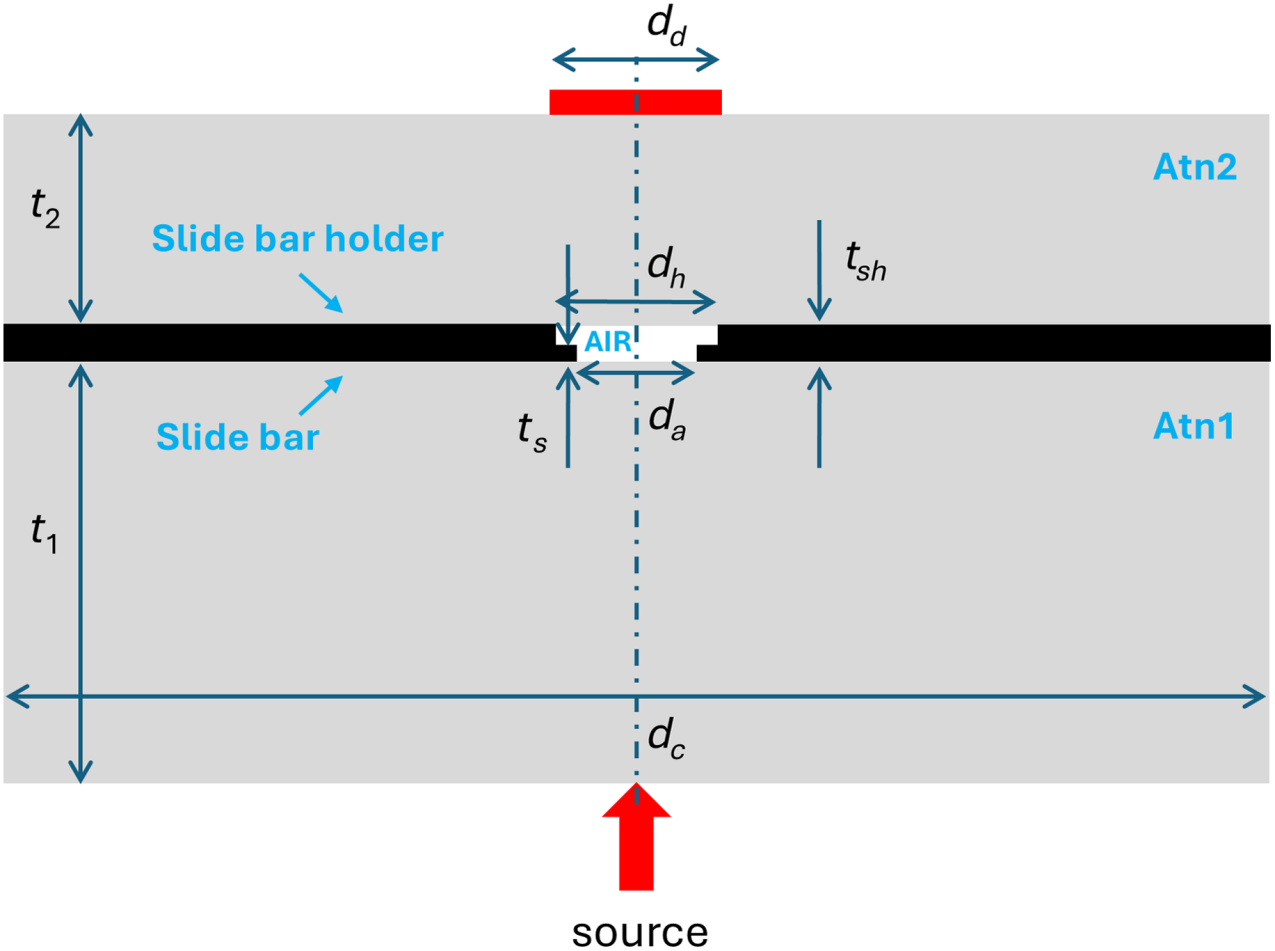
Geometry of the domain for MC simulations. Atn1 and Atn2: cylindrical attenuators; $t_{1}$ and $t_{2}$: thickness of Atn1 and Atn2; $d_{c}$: diameter of Atn1 and Atn2; $t_{s}$: thickness of slide bar; $d_{a}$: diameter of aperture in the slide bar; $d_{h}$: diameter of aperture in the slide bar holder; $t_{sh}$: distance between Atn1 and Atn2; $d_{d}$: diameter of the detector.

Numerical simulations were performed by the MC method using the MMCLAB OpenCL mesh-based MC tool.^12^ The domain (see Fig. 1) was designed to reproduce the real geometry of the fNIRS phantom (see also Fig. 1 in the part 1 paper^9^). The geometry consists of two cylindrical attenuators (Atn1 and Atn2, with thickness $t_{1}=2\;\mathrm{cm}$ and $t_{2}=1\;\mathrm{cm}$, respectively, and diameter $d_{c}=6\;\mathrm{cm}$), a slide bar (with $t_{s}=0.05\;\mathrm{cm}$ thickness) containing an aperture with diameter $d_{a}=0.6\;\mathrm{cm}$, and a slide bar holder containing an aperture with diameter $d_{h}=0.8\;\mathrm{cm}$. The distance between Atn1 and Atn2 is $t_{sh}=0.12\;\mathrm{cm}$. The empty space between the two cylindrical attenuators is filled with air. The mesh’s smallest edge was set to 0.05 cm to enhance geometrical accuracy while maintaining reasonable computational and memory requirements.

Simulations were performed in transmittance geometry, mimicking the source as a pencil beam, and the detector as a disk with diameter $d_{d}=0.8\;\mathrm{cm}$. Each simulation was performed by injecting $10^{9}$ photons. The total number of photons detected ($N_{d}$) through the 8 mm diameter aperture was used to calculate the optical loss ($OL=-\log_{10}(N_{d}/N_{i})$, in dB). Five wavelengths (650, 700, 750, 800, and 850 nm) were simulated. The input optical properties of the cylindrical attenuators Atn1 and Atn2 are reported in Table 1 (derived from the broadband time domain diffuse reflectance measurements, see Sec. 3.2). The optical properties of the slide bar and slide bar holder were assumed as $\mu_{a}=10,000\;{\mathrm{cm}}^{-1}$, $\mu_{s}^{\prime }=100\;{\mathrm{cm}}^{-1}$, with refractive index 1.0. The optical properties of air were assumed as $\mu_{a}=0\;{\mathrm{cm}}^{-1}$, $\mu_{s}^{\prime }=0\;{\mathrm{cm}}^{-1}$, with a refractive index of 1.0. These parameters were kept fixed for all wavelengths. In the simulations, the $\mu_{a}$ was initially set to $0\;{\mathrm{cm}}^{-1}$, and added *a posteriori*, decreasing the photon weight according to the path-length traveled by the photon in each compartment.^13^

**Table 1:** Optical properties of the cylindrical attenuators Atn1 and Atn2 in the MC simulations. Data for $\mu_{a}$ and $\mu_{s}^{\prime }$ were taken from Fig. 2(b).

λ (nm)	$\mu_{a}$ (${\mathrm{cm}}^{-1}$)	$\mu_{s}^{\prime }$ (${\mathrm{cm}}^{-1}$)	n
650	0.002	10.6	1.5
700	0.002	9.9	1.5
750	0.007	9.4	1.5
800	0.006	8.8	1.5
850	0.005	8.4	1.5

Starting from the initial configuration, different simulations were repeated by varying: (a) the diameters of the cylinders: $d_{c}=4\;\mathrm{cm}$, and 8 cm; (b) the thickness of Atn1 (or the position of the internal aperture at the center of the slide bar): $t_{1}=1.0\;\mathrm{cm}$, and 1.5 cm (keeping fixed the total thickness of Atn1 and Atn2 $t_{1}+t_{2}=3\;\mathrm{cm}$ and the distance between Atn1 and Atn2 $t_{\mathrm{sh}}=0.12\;\mathrm{cm}$); (c) the diameter of the internal aperture at the center of the slide bar: $d_{a}=0.4\;\mathrm{cm}$, and 0.8 cm diameter; (d) the size of the incident beam using a uniform disk with 0.5 cm diameter; (e) the bulk absorption and reduced scattering coefficients. Simulations were performed on a Windows 10 Pro workstation equipped with 2 processors of 12 cores at 2.3 GHz, and a GPU NVIDIA GeForce RTX 3080 Ti. The time needed for a simulation was ∼80  s.

### 3.4 Phantom Matrices for Extended Spectral Characterization

BioPixS manufactured a phantom suitable for use in the fNIRS standard based on the same concept as the AIST phantom. BioPixS started from a large block of POM-C material,^14^ 50 mm in thickness. Sheet-extruded POM-C was used because it provides uniform optical properties across the lateral dimensions of the material. This block was then machined down to create uniform samples of thickness 20 and 10 mm, as required by the fNIRS standard. The 20 mm thick block was used for characterizing the optical properties. The samples were then assembled into a 3D-printed casing to create the BioPixS fNIRS standard phantom. The sliding aperture is created using 3D printing of PLA material (Prusa, PLA Prusament Jet black, Prague, Czech Republic) with high carbon black concentration. The input and output sides of the fNIRS standard phantom are designed to permit the attachment of a wide array of optodes.

Silicone was considered an alternative material for the phantom construction (POLIMI). ${\mathrm{TiO}}_{2}$ was added to Silicone Elastomer Sylgard 184 (Base 10 and Curing Agent 1, Dow Corning), for a final concentration of 0.1% (in weight). The mixture was stirred for 3 min and homogenized for 2 min before adding the curing agent (10%). Again, following 2-min stirring and 1-min homogenizing, air bubbles were removed from the solution by a vacuum pump system. Curing was obtained by keeping the samples in the oven (UT 20P, Heraeus, Hanau, Germany) at 65°C overnight. The phantom was cast in a similar shape as the AIST phantom attenuators, namely, a 6 cm diameter, 2.2 cm height cylinder. More details and procedures are reported in detail elsewhere.^10^

## 4 Spectral Characterization of the AIST Phantom

**Fig. 2:**
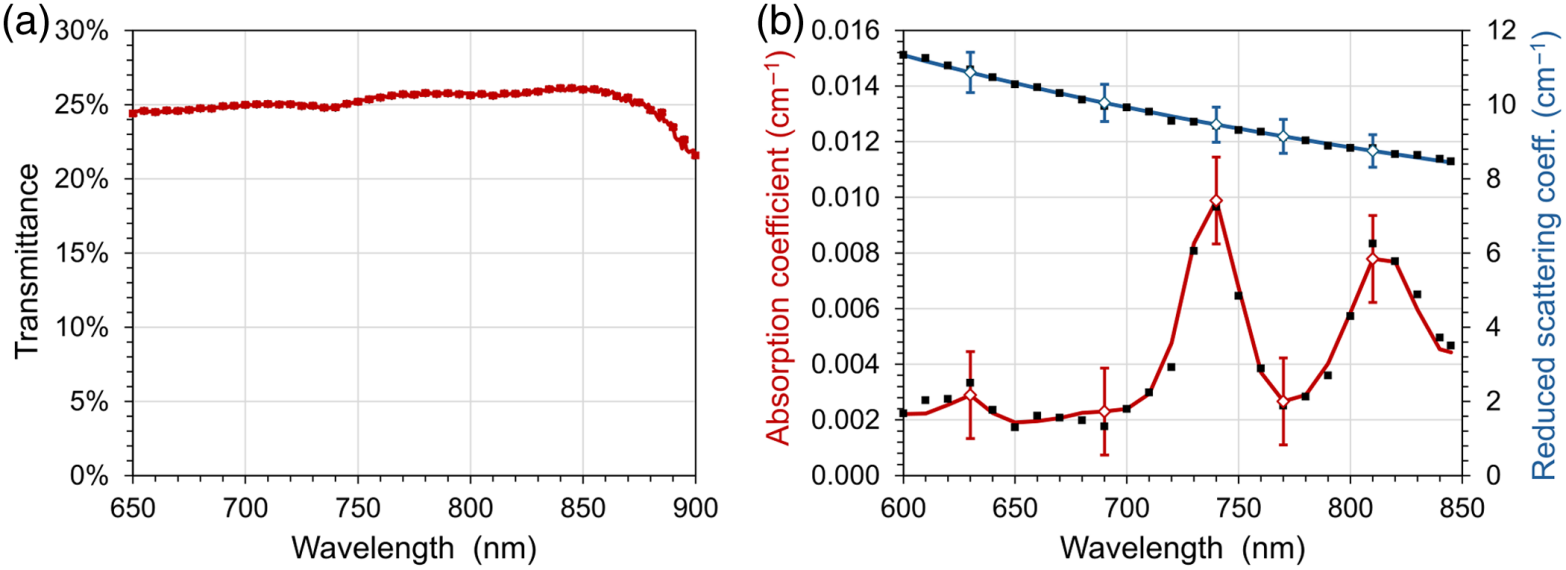
(a) Diffuse transmittance of a 5 mm thin slice of the POM-C matrix from the same batch of the AIST phantom was measured using a spectrometer equipped with an integrating sphere in transmission geometry. (b) Spectra of $\mu_{a}$ (red, left) and $\mu_{s}^{\prime }$ (blue, right) of the 2 cm thick AIST phantom matrix measured in transmittance geometry. The lines indicate the final (smoothed) results when using Eq. (1) for $\mu_{s}^{\prime }(\lambda )$. The solid squares show the underlying raw data. Error bars are plotted for selected wavelengths.

Figure 2(a) shows the transmission spectrum of a thin (2 mm) slice of POM-C material taken from the same batch as used for the AIST Phantom and measured using a standard spectrometer equipped with an integrating sphere as described in Sec. 3.1. Diffuse transmittance of light is largely uniform over the whole 600 to 850 nm spectral range, with only very feeble valleys around 740 and 815 nm. Above 850 nm, the attenuation increases, hinting at a more prominent absorption peak (localized at 900 nm, data not shown). The spectrum shows a slight increase in transmittance with wavelength (10% over 200 nm).

Figure 2(b) shows the absorption and the reduced scattering coefficients of the 2 cm thick POM-C cylinder of the AIST phantom between 600 and 850 nm measured at PTB. Below 700 nm, the absorption is $∼0.002\;{\mathrm{cm}}^{-1}$. At 740 and 815 nm, the absorption spectrum shows two peaks, whereby the maxima do not exceed $0.01\;{\mathrm{cm}}^{-1}$. These peaks are responsible for the small dips in the transmission spectrum in Fig. 2(a). Overall, the absorption is significantly lower than that of most types of biological tissue. The $\mu_{s}^{\prime }$ is within the general range of values for tissue. The decrease with increasing wavelength is comparatively flat. The decay coefficient b in Eq. (1) amounts to ∼0.86. The application of Eq. (1) leads to only minor corrections of $\mu_{s}^{\prime }$ and $\mu_{a}$ compared with the raw data. The uncertainty of the optical properties is dominated by the influence of the refractive index, as addressed in Sec. 7.

## 5 Monte Carlo Simulations

**Fig. 3:**
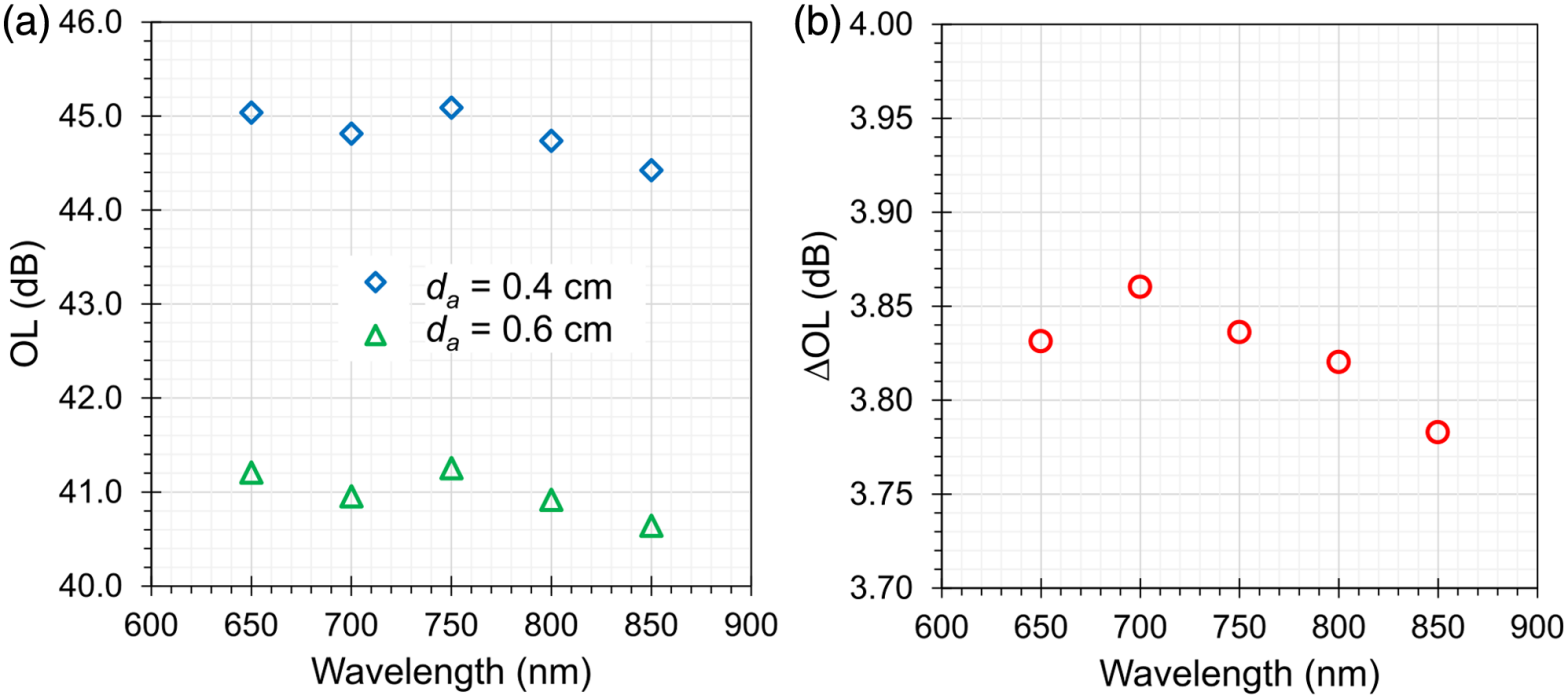
(a) Optical loss of the simulated phantom as a function of wavelength for different internal aperture diameters $d_{a}=0.4$, 0.6 cm. (b) Change in optical loss when changing the internal aperture diameter from $d_{a}=0.6\;\mathrm{cm}$ to $d_{a}=0.4\;\mathrm{cm}$.

The effect of the phantom geometry and optical properties on OL as well as on ΔOL—that is, the change in OL for switching the internal aperture in the slide bar—was studied using MC simulations. Figure 3(a) shows OL as a function of wavelength and for a pencil beam injection with different sizes of the internal aperture $d_{a}$. The estimated OL for the configuration with $d_{a}=0.6\;\mathrm{cm}$ diameter of the internal aperture (green triangles) is between 40.6 and 41.3 dB. A larger OL from 44.4 to 45.1 dB is obtained if the diameter of the internal aperture is reduced to $d_{a}=0.4\;\mathrm{cm}$, as expected. Conversely, a larger internal aperture with $d_{a}=0.8\;\mathrm{cm}$ results in lower OL between 38.3 and 38.9 dB (data not shown). Switching the aperture diameter from $d_{a}=0.6\;\mathrm{cm}$ to $d_{a}=0.4\;\mathrm{cm}$ increases the OL by ∼3.8  dB as shown in Fig. 3(b). Increasing the diameter of the aperture from $d_{a}=0.6\;\mathrm{cm}$ to $d_{a}=0.8\;\mathrm{cm}$ decreases the OL by ∼2.4  dB (data not shown).

**Fig. 4:**
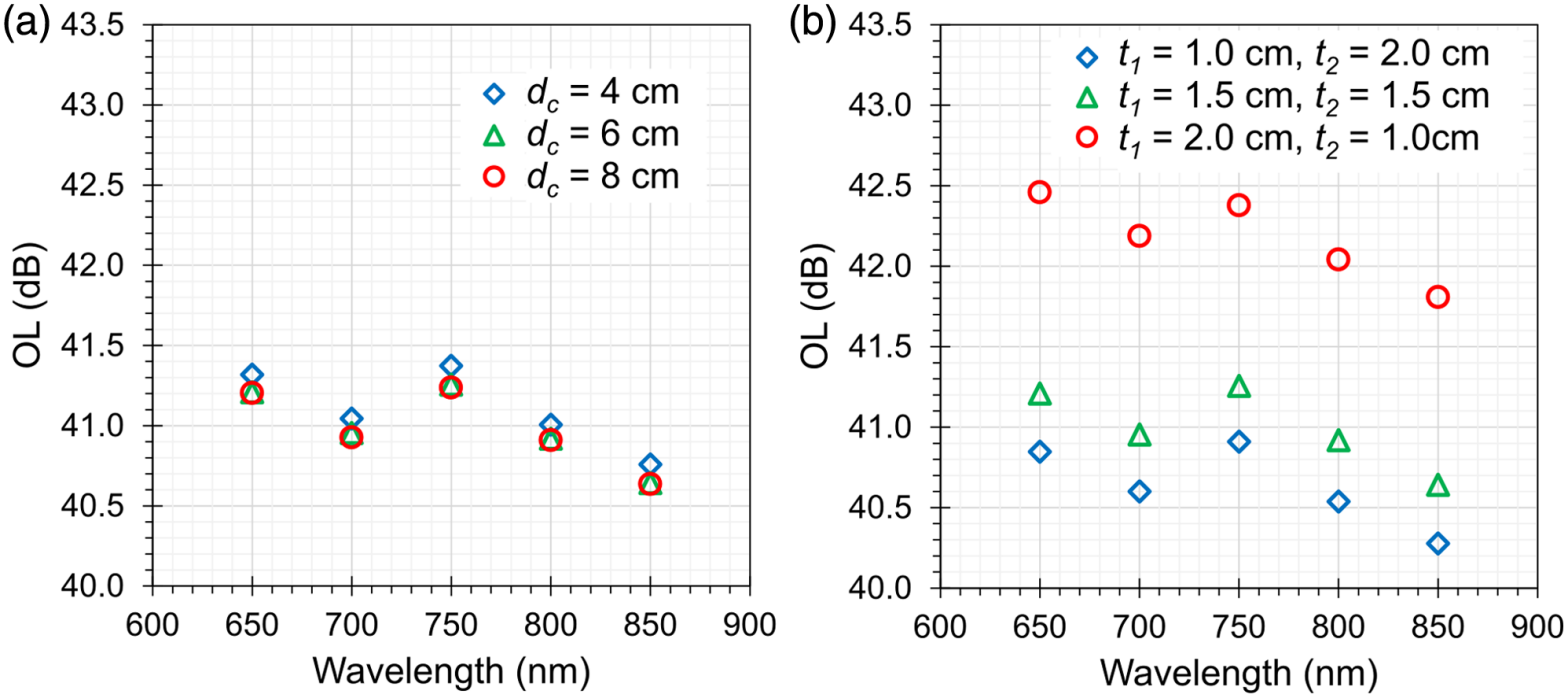
Optical loss of the simulated phantom as a function of wavelength for (a) different cylinder diameters $d_{c}=4$, 6, 8 cm and (b) different positions of the internal aperture $t_{1}=1.0$, 1.5, 2.0 cm while keeping $t_{1}+t_{2}=3\;\mathrm{cm}$ (b).

Changing the diameter of the cylinder has a negligible effect on the OL. Reducing the diameter of the cylinders from $d_{c}=6\;\mathrm{cm}$ to $d_{c}=4\;\mathrm{cm}$ yields an increase in OL of ∼0.1  dB, whereas increasing the diameter from $d_{c}=6\;\mathrm{cm}$ to $d_{c}=8\;\mathrm{cm}$ leads to a maximal decrease of OL by 0.03 dB [see Fig. 4(a)]. The latter (former) results are consistent with an increased (decreased) number of photons lost at the lateral boundary of the cylinder.

To study the effect of the distance of the internal aperture from the source, we have changed $t_{1}$ and $t_{2}$, the thicknesses of cylindrical attenuators Atn1 and Atn2, while keeping fixed the overall thickness $t_{1}+t_{2}=3\;\mathrm{cm}$ and $d_{a}=0.6\;\mathrm{cm}$. The change in OL ranges from −0.35 to −0.38  dB when moving from the configuration with $t_{1}=1\;\mathrm{cm}$ and $t_{2}=2\;\mathrm{cm}$ to that with $t_{1}=2\;\mathrm{cm}$ and $t_{2}=1\;\mathrm{cm}$, whereas it ranges from 1.12 to 1.35 dB for the configuration with $t_{1}=t_{2}=1.5\;\mathrm{cm}$ [see Fig. 4(b)].

**Fig. 5:**
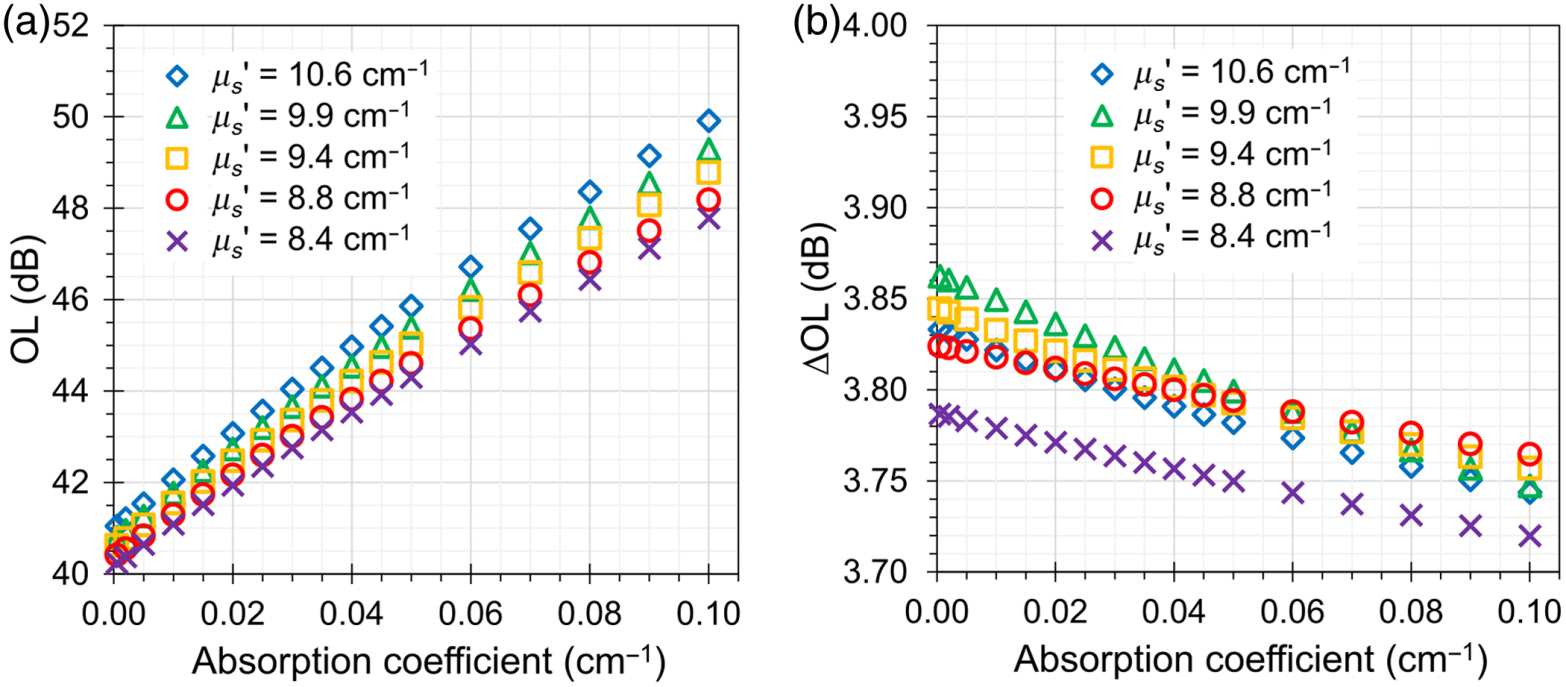
(a) Optical loss of the simulated phantom for different values of the bulk $\mu_{a}$ and bulk $\mu_{s}^{\prime }$ of the cylindrical attenuators Atn1 and Atn2. (b) Change in optical loss of the simulated phantom when the internal aperture is reduced from $d_{a}=0.6\;\mathrm{cm}$ to $d_{a}=0.4\;\mathrm{cm}$ as a function of wavelength for different values of the bulk $\mu_{a}$ and bulk $\mu_{s}^{\prime }$ of the cylindrical attenuators Atn1 and Atn2.

Figure 5(a) systematically shows that the OL—as expected—is influenced by the bulk $\mu_{a}$ of the cylindrical attenuators Atn1 and Atn2. In the range for the bulk $\mu_{a}$ 0.0005 to $0.1\;{\mathrm{cm}}^{-1}$ (a 200× change), the OL increases from ∼40  dB up to 50 dB. However, the change in OL when passing from the internal aperture diameter $d_{a}=0.6\;\mathrm{cm}$ to $d_{a}=0.4\;\mathrm{cm}$ varies less than −0.1  dB when the bulk $\mu_{a}$ spans the range 0.0005 to $0.1\;{\mathrm{cm}}^{-1}$, as shown in Fig. 5(b). The effect of $\mu_{s}^{\prime }$ on the OL is minimal when the absorption is low (<1  dB differences between minimum and maximum $\mu_{s}^{\prime }$ values at $\mu_{a}=0.0005\;{\mathrm{cm}}^{-1}$) and becomes more evident at larger absorption values (<2.1  dB differences between minimum and maximum $\mu_{s}^{\prime }$ values at $\mu_{a}=0.1\;{\mathrm{cm}}^{-1}$).

**Fig. 6:**
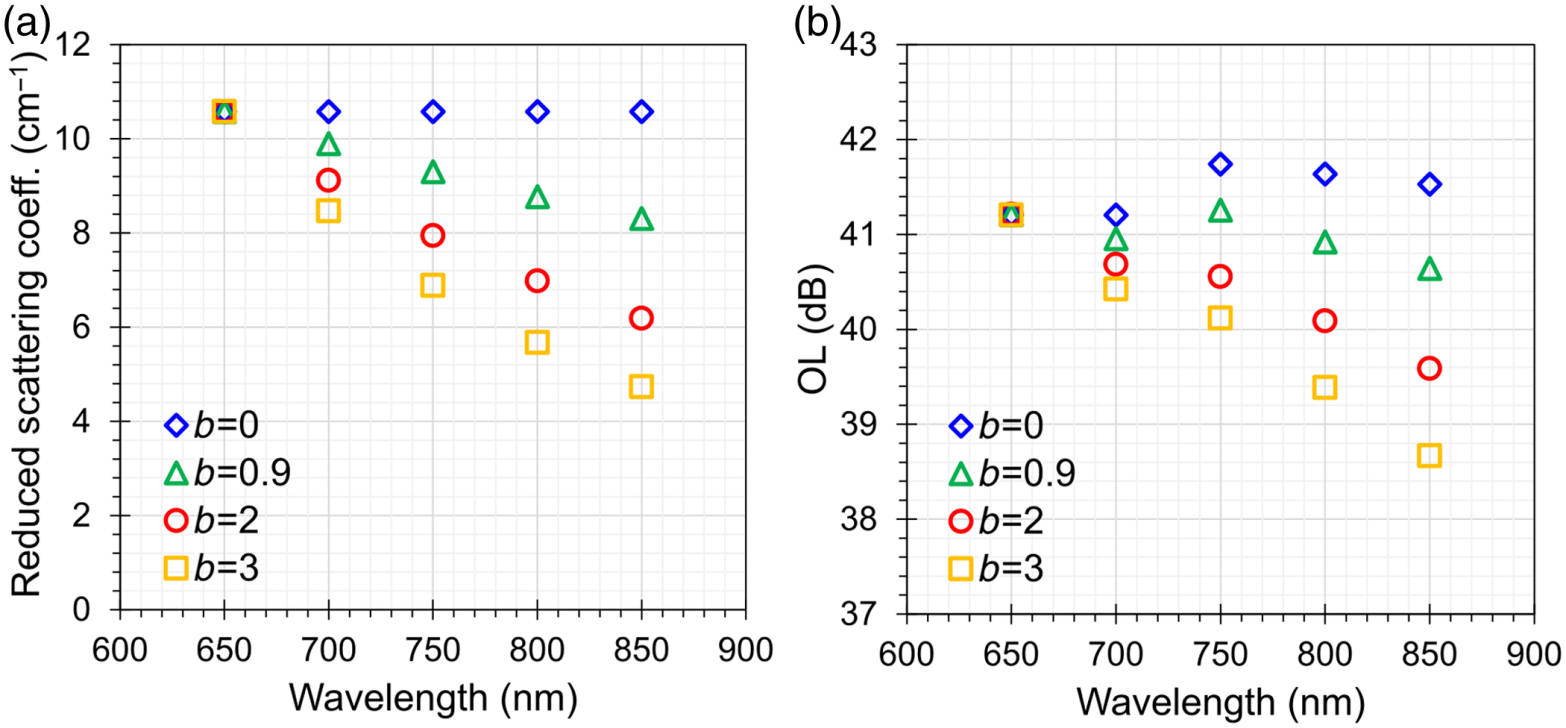
(a) Different spectra of the bulk $\mu_{s}^{\prime }$ of the cylindrical attenuators Atn1 and Atn2. Equation (1) was used keeping fixed $\mu_{s}^{\prime }(\lambda_{0})=10.6\;{\mathrm{cm}}^{-1}$ and $\lambda_{0}=650\;\mathrm{nm}$ while changing b. (b) The corresponding optical loss of the simulated phantom for the different spectra of the bulk $\mu_{s}^{\prime }$ of the cylindrical attenuators Atn1 and Atn2 shown in panel (a).

Figure 6(b) shows the OL for different spectra of $\mu_{s}^{\prime }$ each characterized by a different b parameter, as described in Fig. 6(a). As expected, a larger b corresponds to steeper spectral dependence, resulting in a reduced OL for longer wavelengths.

Finally, the results are not affected if the pencil beam is replaced by a 0.5 cm-diameter uniform beam (data not shown).

## 6 Extended Spectral Characterization of Various Materials for Phantom Construction

The material (POM-C) used for the construction of the AIST phantom has the great advantage of providing intrinsically a $\mu_{s}^{\prime }$ similar to what is observed in biological tissues in the 650 to 900 nm range. For that reason, it was used at the early stages of diffuse optics as a viable phantom material.^15^ Yet, the very fact that POM is naturally scattering makes the exact definition of optical properties less reliable as they depend on the blend and fabrication, different from lab-made phantoms, where absorbers and scatterers can be mixed in due quantities to yield planned properties. In addition, different extrusion and processing of POM-C could provide different optical properties and potential heterogeneity within the material. For example, cylindrical extrusion of POM-C seems to lead to a highly scattering region along the center line. Therefore, we investigated different extrusions of POM-C, and also other materials, frequently used in diffuse optics, with fabrication that is well documented and reproducible in the lab.

In addition, we extended the spectral range to 600 to 1100 nm, because some fNIRS instruments are adopting higher wavelengths and because the potential use of 1064 nm to assess oxygenated hemoglobin changes has been proposed.^16^

**Fig. 7:**
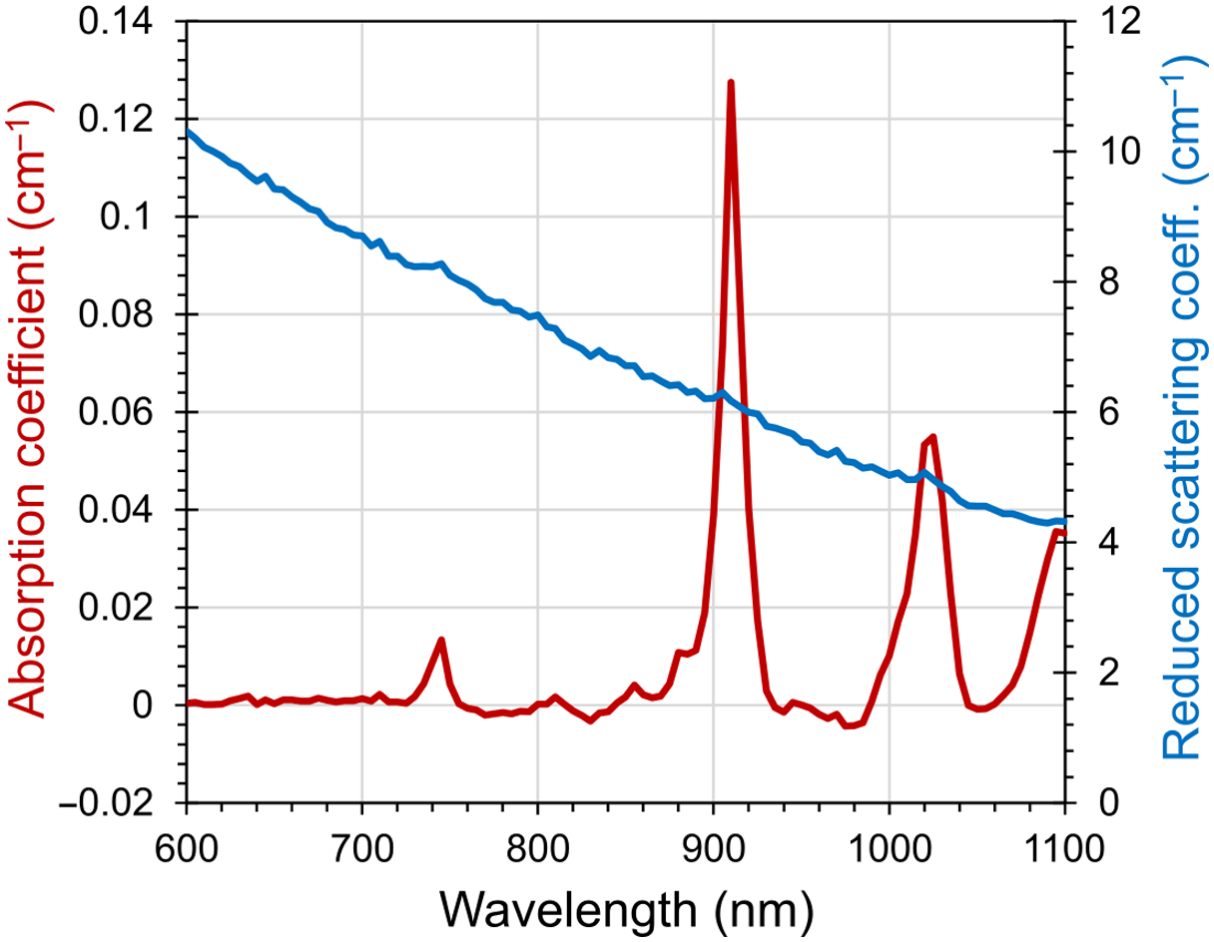
Spectra of $\mu_{a}$ (left axis) and $\mu_{s}^{\prime }$ (right axis) of a Sylgard cylinder [similar dimensions to the AIST phantom in Fig. 2(b): 6 cm of diameter, and 2.2 cm thickness] measured in transmittance geometry.

A first alternative option to POM-C is the use of a silicone (Sylgard) matrix, as described above, with ${\mathrm{TiO}}_{2}$ particles added to control scattering and with no additional absorbers. Figure 7 shows $\mu_{a}$ and $\mu_{s}^{\prime }$ of a silicone matrix with the same dimensions as those of the 2 cm thick block of the AIST phantom. The optical properties of the silicone matrix were measured in the spectral range from 600 nm up to 1100 nm. The absorption is negligible up to 870 nm, apart from a minor peak at 745 nm. Above 870 nm, there are two additional peaks at 910 and 1025 nm. The scattering spectrum is flat and decreasing upon increasing wavelength. As the Sylgard matrix has negligible scattering contributions, the required $\mu_{s}^{\prime }$ values can be finely tuned using ${\mathrm{TiO}}_{2}$ concentration. Compared with the AIST phantom, this matrix shows a steeper scattering spectrum [i.e., a larger b coefficient in Eq. (1): b=1.48].

**Fig. 8:**
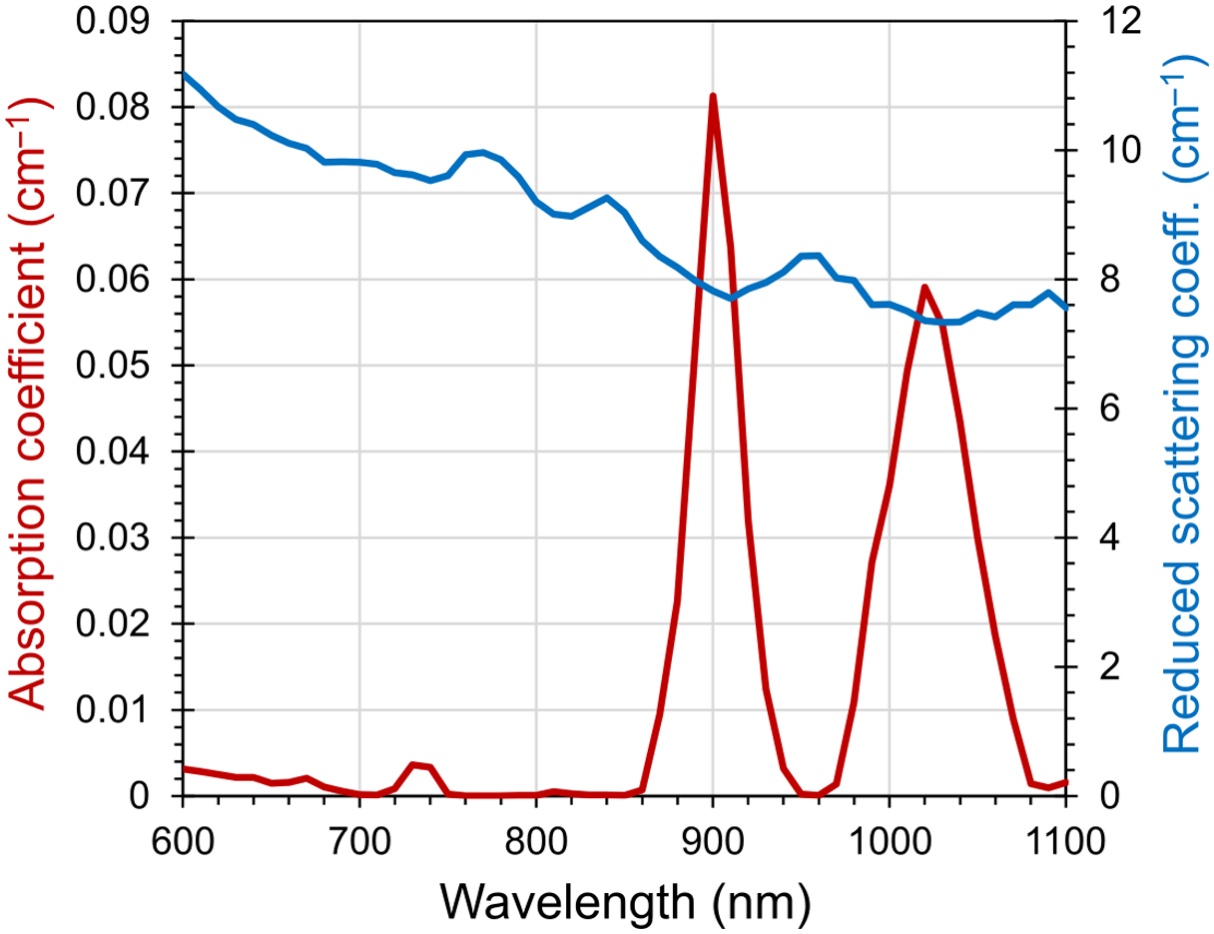
Spectra of $\mu_{a}$ (left axis) and $\mu_{s}^{\prime }$ (right axis) of a BioPixS POM block [thickness 2 cm, i.e., same as the AIST POM block characterized in Fig. 2(b)], measured in transmittance geometry.

Among POM-C materials, the best option is sheet-extruded POM-C, which can provide uniform optical properties across the lateral dimensions of the material. For this purpose, BioPixS adopted sheet-extruded POM-C. For the time domain characterization, a larger block of POM-C material of 50 mm in thickness was machined down to create a sample of 20 mm thickness. Measurements were performed in transmittance geometry. Figure 8 shows the spectral characteristics of the machined POM-C material. The POM-C material exhibits similar scattering properties and negligible absorption, as seen in the AIST POM-C material below 860 nm, whereas above that limit, $\mu_{a}$ increases rapidly with two major peaks at 900 and 1020 nm, respectively.

## 7 Discussion

In general, there is an increasing need to adopt standard procedures and reference phantoms in fNIRS to permit verification of instruments, inter-laboratory comparison, and assessment of clinical studies. In addition, the push for Open Science and deployment of Open Data demands that test procedures and reference data be made available together with experimental data. There are major activities in the development of reference phantoms,^17^ including those for basic testing of devices^18^ and those designed to mimic complex biological structures,^19^^–^^21^ performance assessment procedures,^22^^–^^24^ standardization of analysis,^25^^–^^27^ and inter-laboratory comparison.^28^ This paper will not go into detail on these different approaches and solutions: we will rather focus on the specific requirements of the phantom for the fNIRS standard.

The spectral characterization of the AIST phantom revealed a very low absorption—apart from two minor peaks at 740 and 815 nm, which do not exceed $0.01\;{\mathrm{cm}}^{-1}$—and an almost flat scattering spectrum around $10\;{\mathrm{cm}}^{-1}$ with a Mie-related decay coefficient b in Eq. (1) of ∼0.86. These two properties make the total optical loss OL and even more its change ΔOL to be largely wavelength independent. This specific feature is not strictly required by the standard because manufacturers are instructed to characterize ΔOL using a power meter for each wavelength of the particular fNIRS device, to use this information when calculating the expected changes in deoxygenated and oxygenated hemoglobin. Nevertheless, wavelength independence is a useful condition for a fair application to different devices relying on distinct choices of wavelengths so as to test them under similar attenuation conditions.

Measuring the very small absorption coefficients of the 2 cm thick POM phantom is difficult because they have only little impact on the shape of the DTOF. The results are strongly dependent on boundary effects under these conditions. The uncertainty of the optical properties is dominated by the influence of the refractive index. The data in Fig. 2(b) were obtained by assuming the refractive index of POM as n=1.5 (not specified by the manufacturer), which is a typical value for plastics in the near-infrared spectral range. A change of n of ±0.01 in the MC model shifts the fitting results for $\mu_{a}$ by about $\pm 0.0005\;{\mathrm{cm}}^{-1}$, which corresponds to a relative change in $\mu_{a}$ of ∼20% in the 600 to 700 nm range. The associated change in the results for $\mu_{s}^{\prime }$ is $∼\pm 0.1\;{\mathrm{cm}}^{-1}$, which corresponds to a relative change in $\mu_{s}^{\prime }$ of only ∼1%. The error bars in Fig. 2(b) reflect an assumed uncertainty in the refractive index of ±0.04. Published values of the refractive index for POM are smaller (n=1.435 to 1.414 from 650 to 800 nm).^29^ However, these values were obtained from films of POM-C pressed from granulate. These films could contain inclusions of air being responsible for a reduction of the observed refractive index. If these smaller values were adopted in the fitting procedure based on the MC model, $\mu_{a}$ would become negative and therefore unrealistic. This observation supports the assumption that the refractive index must be higher for the extruded POM used in the AIST phantom than the values reported for the pressed granulate. To reduce the uncertainty of $\mu_{a}$, the refractive index of POM-C should be measured. A suitable method appears to be the determination of the Brewster angle based on reflection measurements on a flat, polished surface, as was done in Ref. 29 for the pressed granulate. The fNIRS standard does not contain any specifications regarding the refractive index of the phantom material. It defines a range for $\mu_{s}^{\prime }$ and $\mu_{a}$ to ensure optical properties similar to those of brain tissue. However, a model-based determination of the scattering and absorption properties requires knowledge of the refractive index.

As discussed in the companion paper part 1,^9^ there is a wide variety of white POM bulk products available. For the fabrication of a standard phantom, the reproducibility in the optical properties, the optical homogeneity, and the surface roughness are relevant features to be considered. For instance, “center line porosity” is a known issue in POM-H (polyoxymethylene homopolymer) such as Delrin^®^ and should be avoided. Conversely, POM-C materials as Duracon were observed to provide a good level of optical homogeneity and were selected for the AIST phantom, as well as for the BioPixS phantom.

The MC simulations permitted to identify some remarkable properties of the designed phantom. The spectral variations in OL are limited due to the rather flat scattering and very low absorption, with an increase of 0.7 dB from 650 to 800 nm due to a small absorption peak in POM. The corresponding variation in ΔOL is negligible (<0.08  dB). Furthermore, though the phantom is rather small (just 6 cm in diameter), no significant finite-size effect is observed. Indeed, OL remains unchanged for a larger diameter (8 cm) and is unaltered for an even smaller size (4 cm). It is still true—as commented above—that within the bulk materials, key losses are caused by boundaries for $\mu_{a}\approx 0\;{\mathrm{cm}}^{-1}$, but with the insertion of the black plate with the hole in between the two attenuators, the main light losses are governed by the aperture geometry rather than by the lateral boundaries of the phantom.

In agreement with this design, ΔOL shows only a weak dependence on $\mu_{a}$ within the explored range (0 to $0.5\;{\mathrm{cm}}^{-1}$), remaining confined within 3.5 to 3.8 dB, well inside the 3 to 4 dB limits prescribed by the standard. This behavior is consistent with the fact that ΔOL is primarily determined by the ratio of the aperture areas in the black plate.

In addition, the position of the black plate along the axis of the phantom is not so critical, with just <1  dB difference when the plate is placed in the middle of the phantom or with a displacement of 0.5 cm from the center (i.e., the actual realization, with 2 and 1 cm thickness, respectively).

MC simulations further confirm that ΔOL is very close to the ratio of the areas of the apertures, at least up to the largest tested diameter of 0.8 cm. The simulated OL at 700 nm was 42 dB, slightly different from the measured value of 40 dB. At this level, inaccuracies in the estimate of the optical properties, in the modeling of the complex geometry of the plate with the slider, or the nonideal surface roughness could create the discrepancy. The 40 dB upper limit for attenuation as prescribed in the standard could be too low to replicate the effective attenuation observed on the human head when dealing with large source-detector distances. The addition of a 20 dB neutral density filter can raise the OL to 60 dB, as discussed in part 1.^9^ It is also possible to increase OL by adding absorption in a fabricated phantom matrix. As shown from MC simulations, increasing $\mu_{a}$ up to $0.5\;{\mathrm{cm}}^{-1}$ raises OL from 40 to 70 dB. ΔOL is rather independent from $\mu_{a}$, being confined in the 3.5 to 3.8 dB range over the explored range from 0 to $0.5\;{\mathrm{cm}}^{-1}$ of $\mu_{a}$, well within the 3 to 4 dB limits prescribed by the standard.

The phantom can be constructed with alternative materials, such as silicone. Example spectra of a Sylgard matrix with no added absorption are reported in Sec. 6. As compared to the AIST phantom, these materials exhibit a more steeply descending scattering spectrum (b=1.48 against b=0.83 of POM). As shown in Sec. 5, the greater slope in scattering increases the wavelength dependence of OL, whereas ΔOL is substantially unaffected. As discussed above, wavelength independence is not a requirement of the standard, but rather a helpful attribute that facilitates fair comparisons between instruments operating at different wavelengths. The adoption of such materials is advantageous because it permits fine adjustment of optical properties, with the addition of black toner for absorption and ${\mathrm{TiO}}_{2}$ particles for scattering. This can be useful—for instance—to yield a 60 dB OL. In addition, good homogeneity and reproducibility can be assured. However, reliable phantom fabrication is a critical task that requires mastery; therefore, Duracon POM-C remains a good practical solution.

The AIST phantom, implementing the fNIRS standard, can be operated only in transmittance geometry. As some devices might not permit detachment of source from detector (e.g., in many biomedical optical applications, sources and detectors are integrated within a single probe to ensure a fixed source–detector separation and stable optical coupling), a phantom operated in reflectance geometry is advisable. However, keeping ΔOL constant independently of source-detector distances is not trivial. There is certainly room for further research on this issue and potential expansion of the standard. In addition, the present standard neither addresses depth sensitivity nor time- or frequency-domain instruments. In a future revision of the standard, such expansion could be desirable, implying updates to test phantom designs.

## 8 Conclusion

The international standard for fNIRS devices, IEC 80601-2-71, foresees specific tests based on a 3 dB to 4 dB change in a dynamic phantom with a baseline 40 dB optical loss in the 650 to 850 nm range. A companion paper^9^ describes the AIST phantom, which implements the requirements of the standard, and presents the characterization of its attenuation properties as well as its practical application in tests of commercial devices.

In the present paper, we provide the spectral characterization in the 600 to 850 nm range of the turbid materials of the first prototypes of such a phantom produced in Japan by AIST. The absorption coefficient of this POM-C material is extremely low, with minor peaks at 740 and 815 nm not exceeding $0.01\;{\mathrm{cm}}^{-1}$. The reduced scattering coefficient is rather flat and monotonically decreasing from 11.5 down to $8.5\;{\mathrm{cm}}^{-1}$. An extended spectral characterization up to 1100 nm of a different slab of POM-C material showed marked absorption peaks at 900 and 1020 nm, which should be considered when operating above 860 nm.

MC simulations confirmed several notable features of the AIST phantom, attributable to its specific optical properties and geometry. In particular, both OL and ΔOL were found to be largely independent of: (i) wavelength within the 600 to 850 nm range; (ii) the lateral dimensions of the phantom cylinders, even for diameters as small as 4 cm; and (iii) the axial position of the black plate, i.e., the relative thickness of the two attenuator cylinders. The MC simulations could be easily extended to reproduce a multi-aperture design and to evaluate the influence of increased spacing between two attenuators due to thick apertures, which are features of the phantom concept.^18^

The AIST phantom was constructed with a POM-C (Duracon) matrix. Alternative materials for phantom production with well-documented provision and fabrication information are also presented. This option permits to finely tune the optical properties with the addition of ${\mathrm{TiO}}_{2}$ for scattering and black toner for absorption. A key benefit could be the option to raise OL to 60 dB, closer to the typical attenuation in real applications of the device. MC simulations demonstrated that $\mu_{a}$ can be increased up to $0.1\;{\mathrm{cm}}^{-1}$ with minor effects on ΔOL still confined in the 3.5 to 3.8 dB range, well within the 3 to 4 dB limits of the standard.

Overall, these data can be useful to identify a suitable phantom for the implementation of the fNIRS standard. In addition, they provide additional information to complement the standard. Furthermore, this work can facilitate the manufacturing of fNIRS standard phantoms by commercial providers of turbid phantoms.

## Data Availability

The data that support the findings of this study are available from the corresponding author upon reasonable request.
